# Complete genome sequence of the multidrug-resistant *Citrobacter portucalensis* strain KOS1-1 isolated from a wastewater treatment plant

**DOI:** 10.1128/mra.01416-25

**Published:** 2026-02-19

**Authors:** Shahjahon Begmatov, Andrey L. Rakitin, Alexey V. Beletsky, Andrey V. Mardanov, Nikolai V. Ravin

**Affiliations:** 1 Institute of Bioengineering, Research Center of Biotechnology of the Russian Academy of Sciences, Moscow, Russia; Rochester Institute of Technology, Rochester, New York, USA

**Keywords:** multidrug-resistant bacteria, *Citrobacter*, plasmid-mediated resistance, wastewater treatment

## Abstract

The emergence of multidrug-resistant bacteria is a major concern from both epidemiological and ecological perspectives. Studying these microorganisms is crucial for developing prevention strategies. We report the complete genome sequence of the multidrug-resistant *Citrobacter portucalensis* strain KOS1-1, isolated from wastewater in the city of Moscow.

## ANNOUNCEMENT

The growing interest in multidrug-resistant (MDR) strains from wastewater treatment plants (WWTP) stems from their role as potential reservoirs of mobile antibiotic resistance genes (ARGs). Consequently, their genetic makeup and resistomes are increasingly being characterized. MDR *Citrobacter* strains have been isolated from WWTP-related samples, and many of them produce carbapenemase and extended-spectrum β-lactamase, and these genes are consistently associated with a wide spectrum of mobile genetic elements (1). MDR bacteria can survive wastewater treatment processes and be released into the environment with the effluent (2). Moscow’s WWTPs, among the largest in the world, process approximately 6 × 10^6^ m^3^ of water per day. Treated wastewater is discharged into the Moscow River, constituting nearly 50% of its total flow. Due to their immense scale, these WWTPs represent an important source for the dissemination of ARGs into the environment (3).

*C. portucalensis* strain KOS1-1 was isolated by plating of untreated influent wastewater on LB agar medium supplemented with tetracycline (20 µg/mL), chloramphenicol (20 µg/mL), kanamycin (50 µg/mL), and ampicillin (50 µg/mL) (4). The strain was grown in LB broth supplemented with these antibiotics at 20°C for 2 days; genomic DNA was extracted from 50 mL of culture using the DNeasy PowerSoil Kit (Qiagen, Germany) according to the manufacturer’s protocol.

A hybrid sequencing approach combining Illumina and Oxford Nanopore platforms was used to sequence the genome. The library for Illumina sequencing was prepared using the NEBNext Ultra II DNA Library prep kit (New England Biolabs, USA). Its sequencing on the MiSeq instrument (Illumina, USA) generated 741,626 read pairs (2 × 300 nt) with a total length of 446.45 Mb. Overlapping reads were merged using FLASH v.1.2.11 (5) with “M 300” parameter, low-quality sequences were trimmed with Sickle v.1.33 (https://github.com/najoshi/sickle) with a quality threshold of *Q*_30_. Additionally, the *C. portucalensis* KOS1-1 genome library was prepared from non-sheared DNA using the 1D Genomic DNA ligation sequencing kit SQK-LSK114 and sequenced in a FLO-MIN114 flow cell using a MinION device (Oxford Nanopore, UK). A total of 17,354 reads with a total length of 276 Mb were obtained. The *N*_50_ value was 23,343 bp, and the average read length was 15,880 bp. Basecalling was computed by Dorado v.0.9.1 (6) using the dna_r10.4.1_e8.2_400bps_sup@v5.0.0 model.

The MinION reads were assembled using Flye v. 2.9.5 (7) with --nano-hq and --iterations 2 parameters. The sequences of the assembled contigs were polished with Illumina reads using Pypolca v.0.3.1 (8). The assembled complete genome was 5,556,809 bp in size and consisted of a chromosome and five circular plasmids. Genes were predicted using NCBI Prokaryotic Genome Annotation Pipeline (version 2023-05-17.build6771) (9). Approximately 99% of Illumina and 88% of MinION reads passed quality filters. Genome statistics are described in Table 1.

**TABLE 1:** Genome sequencing statistics

Parameter	Value
Illumina sequencing	
Read pairs number	741,626
Total bases	446,458,852
Nanopore sequencing	
Reads number	18,901
Total bases	298,186,162
Genome assembly	
Total length, bp	5,556,809
Genome coverage	99.0
Chromosome, bp	4,878,312
Plasmid pKOS1-1-1, bp	77,569
Plasmid pKOS1-1-2, bp	80,667
Plasmid pKOS1-1-3, bp	110,147
Plasmid pKOS1-1-4, bp	121,081
Plasmid pKOS1-1-5, bp	289,033
GC content (%)	51.69
Protein-coding genes	5,227
tRNA genes	85
rRNA genes	25
ncRNA genes	7
Pseudo genes	178

The average nucleotide sequence identity between the genomes of *C. portucalensis* KOS1-1 and the type strain *C. portucalensis* A60^T^ (10), calculated by FastANI v.1.33 (11), was 98.28%, confirming the species assignment of strain KOS1-1. All tools were run with default parameters unless otherwise specified.

The genome contained 14 ARGs that were predicted to confer resistance to β-lactams, quinolones, aminoglycosides, macrolides, sulfonamides, trimethoprim, phenicols, and tetracyclines (4). The complete genome of *C. portucalensis* KOS1-1 facilitates comparative genomic studies of MDR *Citrobacter* strains.

## Data Availability

The whole-genome nucleotide sequence of *C. portucalensis* KOS1-1 has been deposited in DDBJ/ENA/GenBank under accession numbers CP199069, CP199070, CP199071, CP199072, CP199073, and CP199074, and the raw reads have been deposited in the NCBI Sequence Read Archive under accession numbers SRX31239522 and SRX31239521. The version described in this paper is the first version.
